# Muscle contributions to the acceleration of the whole body centre of mass during recovery from forward loss of balance by stepping in young and older adults

**DOI:** 10.1371/journal.pone.0185564

**Published:** 2017-10-25

**Authors:** David F. Graham, Christopher P. Carty, David G. Lloyd, Rod S. Barrett

**Affiliations:** 1 School of Allied Health Sciences, Griffith University, Queensland, Australia; 2 Menzies Health Institute Queensland, Griffith University, Queensland, Australia; Universite de Nantes, FRANCE

## Abstract

The purpose of this study was to determine the muscular contributions to the acceleration of the whole body centre of mass (COM) of older compared to younger adults that were able to recover from forward loss of balance with a single step. Forward loss of balance was achieved by releasing participants (14 older adults and 6 younger adults) from a static whole-body forward lean angle of approximately 18 degrees. 10 older adults and 6 younger adults were able to recover with a single step and included in subsequent analysis. A scalable anatomical model consisting of 36 degrees-of-freedom was used to compute kinematics and joint moments from motion capture and force plate data. Forces for 92 muscle actuators were computed using Static Optimisation and Induced Acceleration Analysis was used to compute individual muscle contributions to the three-dimensional acceleration of the whole body COM. There were no significant differences between older and younger adults in step length, step time, 3D COM accelerations or muscle contributions to 3D COM accelerations. The stance and stepping leg Gastrocnemius and Soleus muscles were primarily responsible for the vertical acceleration experienced by the COM. The Gastrocnemius and Soleus from the stance side leg together with bilateral Hamstrings accelerated the COM forwards throughout balance recovery while the Vasti and Soleus of the stepping side leg provided the majority of braking accelerations following foot contact. The Hip Abductor muscles provided the greatest contribution to medial-lateral accelerations of the COM. Deficits in the neuromuscular control of the Gastrocnemius, Soleus, Vasti and Hip Abductors in particular could adversely influence balance recovery and may be important targets in interventions to improve balance recovery performance.

## Introduction

Older adults fall more frequently compared to their younger counterparts in part because of a reduced capacity to recover from loss of balance [1–3]. Perhaps the most common strategy employed to avoid a fall involves taking a rapid step in the direction of loss of balance. Prospective studies of step recovery performance in older adults demonstrate stepping behaviour measured at baseline is predictive of real world falls experienced over the following 12-month period [4–6]. It therefore follows that a clear understanding of the age-related neuromuscular deficits in stepping ability are required so that targeted interventions to improve stepping reactions may be developed.

Most studies of stepping behaviour in older adults to date have focused on recovery from forward loss of balance, perhaps because trips during walking are a common cause of loss of balance in community dwelling older adults [7, 8]. Studies have demonstrated that successful balance recovery is achieved by taking a rapid and sufficiently long recovery step [3, 9, 10] while simultaneously controlling the rate of forward flexion of the trunk [11–13] and maintaining lateral stability [14]. Older adults that can recover from more severe balance perturbations also have greater lower extremity muscle strength [15, 16] and produce higher joint power [9, 17, 18], and levels of muscle activation in the stepping limb during the recovery step [19]. While these studies provide important information regarding neuromuscular and biomechanical factors associated with successful balance recovery, little is yet known about the cause and effect relationship between muscle force generation and movement patterns during balance recovery.

Determining how the motor system contributes to the control of balance recovery performance is difficult because muscle forces cannot be measured directly or easily. Musculoskeletal models provide a framework whereby the role of muscle forces in the production and control of movement can be investigated [20]. In particular muscle induced acceleration analysis (IAA) may be used to determine the extent to which any given muscle can accelerate any given joint or segment or the body [21], and thereby uncover the coordination strategy used by the neuromuscular system to generate complex movements such as balance recovery by stepping. IAA has been used to describe the contribution of muscle forces to the support and progression of the COM during running [22], normal and pathological gait [23–25] and stair ambulation [26]. Graham et al. [27] used IAA to examine the contributions of individual muscles to the accelerations experienced by the lumbar spine and hip and knee joints on the swing leg during balance recovery in older adults that were able to recover with a single step compared to those that required multiple steps. The main finding was that older adults that required multiple steps used more stance leg hamstring muscle force to generate similar spine and hip accelerations than the single step recovery group. The multiple step balance recovery group was therefore considered to be both less effective and less efficient than the single step balance recovery group. While this study revealed the muscle coordination strategies used to control the stepping leg, it currently remains unknown how individual muscles contribute to the accelerations experienced by the whole-body COM during balance recovery. Such an analysis is important because the conditions for balance recovery can be defined by the requirement to maintain vertical support, while simultaneously controlling the horizontal accelerations experienced by the COM. The muscles that are found to control the COM could subsequently be targeted in exercise-based fall prevention programs. The purpose of this study was to use IAA to determine muscular contributions to the acceleration of the whole-body COM in older compared to younger adults that could recover from forward loss of balance using a single step.

## Materials and methods

### Participants

Fourteen community dwelling older adults aged 65 to 80 years (Age: 72.0 ± 4.8 years, Weight: 82.6 ± 13.1 kg, Height: 1.62 ± 0.10 m) and six younger adults (Age: 28.5 ± 2.0 years, Weight: 78.3 ± 6.4 kg, Height: 1.75 ± 0.10 m) were recruited from the local community. Individuals previously diagnosed with neurological, metabolic, cardio-pulmonary, musculoskeletal and/or uncorrected visual impairment were excluded. Ethics approval was obtained from the Griffith University Human Research Ethics Committee and all relevant ethics guidelines including provision of written informed consent were followed.

### Experimental procedures

The balance recovery protocol was undertaken as reported previously [1, 9, 27]. Participants stood barefoot with their feet shoulder-width apart in an upright posture and were subsequently tilted forward, with their feet flat on the ground, until the required load in body weight (BW) was recorded on a load cell (S1W1kN, XTRAN, Australia) placed in series with an inextensible cable. One end of the cable was attached to a safety harness worn by the participant at the level of their sacrum and the other end was attached to an electric winch on a rigid metal frame located behind the participant. The length of the cable was adjusted until the required force on the cable was achieved. Care was taken to ensure the cable was aligned parallel with the ground and that participants kept their head, trunk and extremities aligned prior to cable release. The cable was released at a random time interval (2–10 s) following achievement of the prescribed posture and cable force (±1%BW), through the disengagement of an electromagnet located in-series with the cable. Participants were instructed to relax their muscles while leaning and to regain balance with a single step using the stepping lower limb of their choice following cable release. The instruction to attempt to recover using a single step was reiterated prior to every trial. A second cable, instrumented with a load cell (S1W1kN, XTRAN, Australia), attached the safety harness to the ceiling, was used to prevent participants from contacting the ground in the event of a failed recovery. Centre of pressure location was displayed in real time on a computer monitor and was visually inspected by the investigator to ensure anticipatory actions (e.g., antero-posterior and medio-lateral weight shifting) were not evident in the period immediately prior to cable release. Following initial familiarisation trials at 15%BW lean angle participants were given a brief rest before completing a single trial at 20% BW. The initial lean angles at the 20% BW lean magnitude relative to vertical were not significantly different between groups (Young: 18.7±1.1°; Old: 18.3±1.9°). Specific events during the stepping phase of balance recovery were defined as follows: Cable release (CR) was identified from a 5 N drop in force measured in the horizontal restraining cable, toe off (TO) was identified from the first vertical motion greater than 2.5 mm of the great toe marker on the stepping foot [28], foot contact (FC) from a force in excess of 5% of the participants body weight recorded on the anterior force plate and the maximum knee joint flexion angle (KJM) from the maximum flexion angle made by the stepping leg flowing foot contact.

Ten older adults and all younger adults recovered with a single step while the remaining four older adults required multiple steps to recover. A multiple step strategy was identified using previously defined criteria [1] as a) a second step of any kind by the stepping limb or progression of the non-stepping limb past the stepping foot following the initial step, b) lateral deviation of the lateral malleolus marker on the non-stepping foot by greater than 20% of body height from its position at cable release and c) if a force of greater than 20% BW was detected in the load cell attached to the ceiling restraint. Of those who took multiple steps two used two or more recovery steps, one applied greater than 20% body weight to the overhead restraining cable and one took a substantial lateral step. Data from the four participants that were unable to recover with a single step was excluded from further analysis. Trajectories of 51 reflective markers attached to each participant [11] and Ground Reaction Forces were recorded simultaneously. Recovery step length was computed using the relative horizontal position of the marker located on the heel of the stepping at cable release to foot contact.

### Computation of muscle induced accelerations

**Fig 1:**
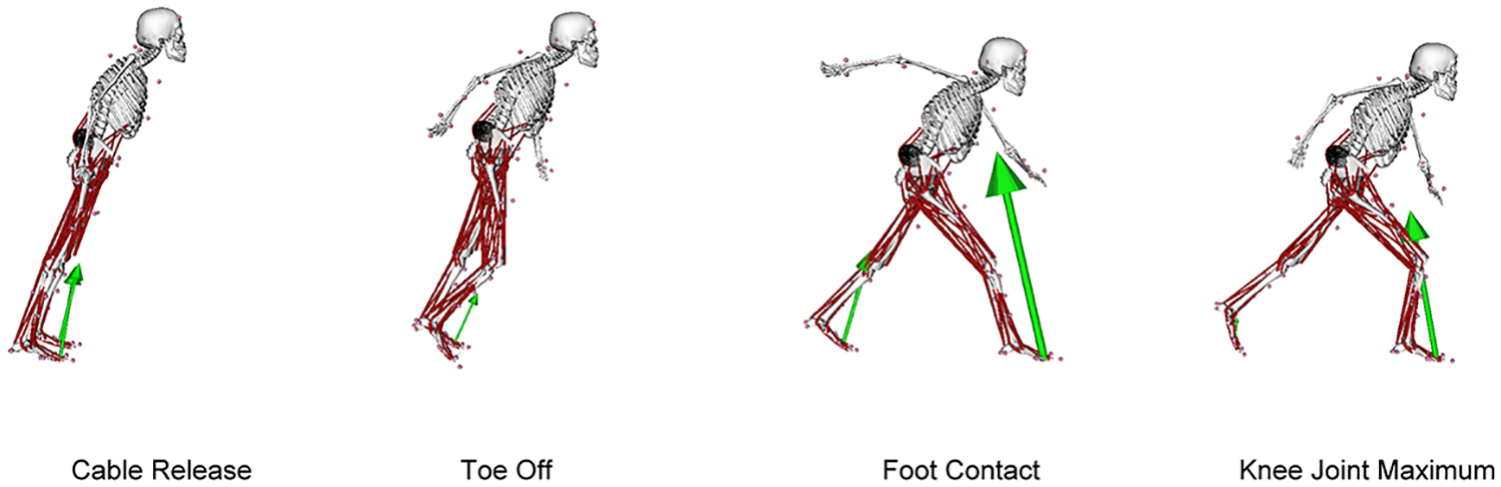
Body configurations at cable release, toe off, foot contact and knee joint maximum during balance recovery for a representative participant. The vectors represent resultant ground reaction forces measure under each foot and the wrap object required to maintain erector spinae moment arms is visible in the region of the lower back.

All data analysis were performed using OpenSim (version 3.2) [20] in conjunction with custom Matlab scripts (Version 2014b, The Maths Works, USA). The musculoskeletal model described by Hamner et al. [29] including 17 bodies (head, torso, pelvis, and bilateral humerus, radius, ulna, hand, femur, tibia, foot) with 17 joints and 36 degrees of freedom (pelvis: 6, neck: 3, lumbar joints: 3, hip: 3, shoulder joints: 3, wrist: 2, elbow: 1, radioulnar: 1, knee: 1, ankle: 1) was used as generic scalable model (Fig 1). 92 hill-type muscle actuators were used to actuate the lumbar and lower extremity joins while the arms were driven by torque actuators. The mass of the harness worn during balance recovery trials was added to the model as a component of the total mass of the participant. A wrap object was embedded in the generic model as previously reported [27] to achieve erector spinae muscle moment arms during trunk flexion consistent with previous reports [30]. Model scaling and inverse kinematic analysis (IK) [31] were performed by fitting the anatomical model to measured 3 dimensional (3D) marker positions with a high weighting on virtual markers which defined the joint centre of the hip, knee and ankle. Joint centres were estimated from experimental marker trajectories: the regression equations of Harrington et al. [32] were used for the hip joint (as suggested by Kainz et al. [33]), while the knee and ankle joint centres were identified as the midpoints of the femoral condyles and the medial and lateral malleoli respectively. Residual Reduction Analysis (RRA) was subsequently performed to improve the dynamic consistency between measured ground reaction forces and the mass-acceleration product of the model [20]. The Static Optimisation tool in OpenSim was used to calculate muscle forces using a cost function that minimised the sum of squared muscle activations within the force-length-velocity constraints of each muscle. Induced Acceleration Analysis was subsequently performed to determine the contribution of each muscle force to the vertical and horizontal accelerations of the whole body COM. For reporting purposes muscle actuators were grouped as follows: Erector Spinae, Gluteus Maximus, Iliopsoas (psoas and iliacus), Rectus Femoris, Hip Abductors (gluteus medius and minimus), Vasti (vastus lateralis, intermedius and medius), Hamstrings (biceps femoris, semimembranosus and semitendonosus), Tibialis Anterior, Gastrocnemius (medial and lateral gastrocnemius) and Soleus. The full length of trials was taken as the period from cable release (CR) to the maximum knee joint angle made by the stepping leg following foot contact (KJM). All analysis were conducted over 3 phases of the balance recovery task which were defined as described previously [1]: Cable Release to Toe Off (CR-TO); Toe Off to Foot Contact (TO-FC); and Foot Contact to the maximum knee flexion angle (Knee Joint Maximum) following foot contact (FC-KJM). Recovery step length was computed using the relative horizontal position of the marker located on the heel of the stepping leg at cable release to foot contact.

### Model evaluation

**Fig 2:**
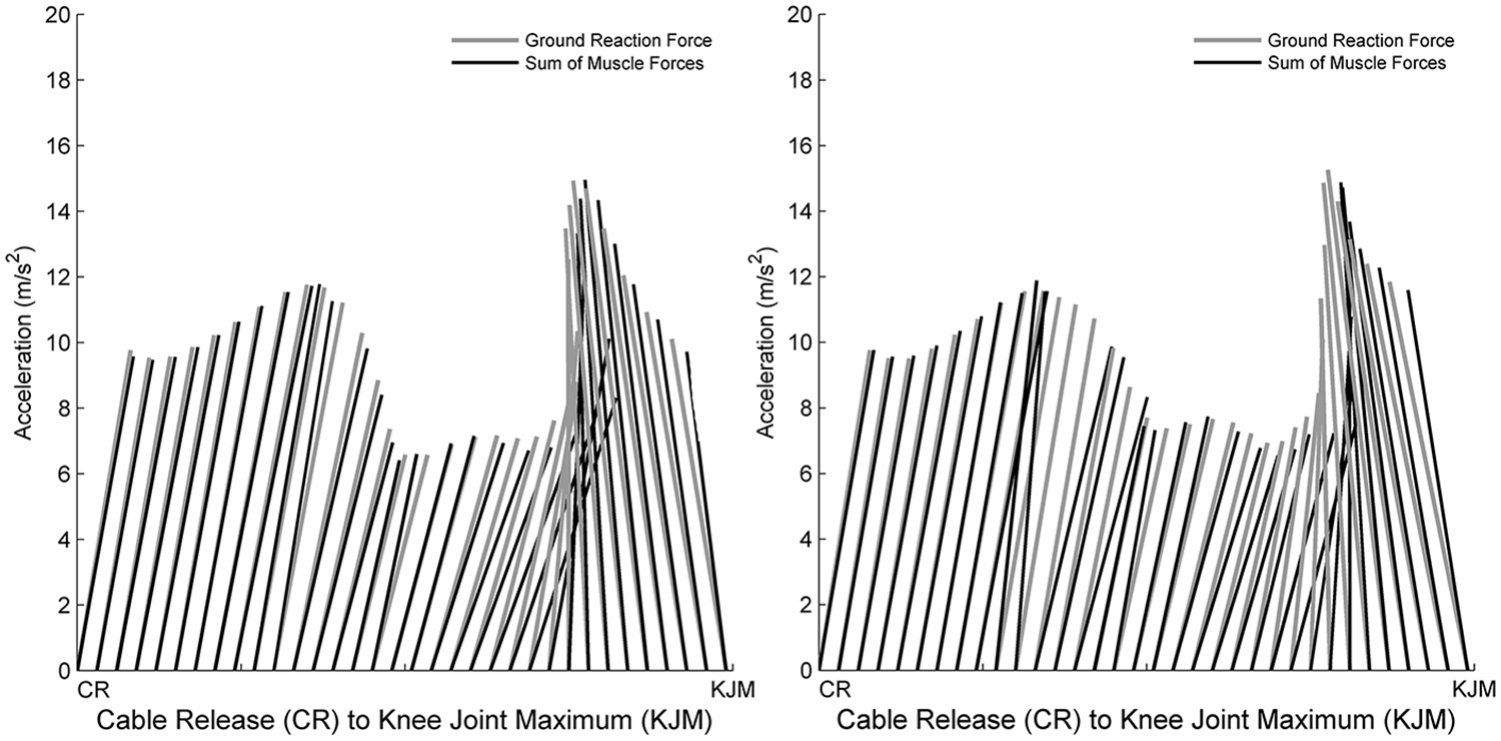
Vector plots for older adults (A) and younger adults (B) representing the acceleration induced by the experimentally measured ground reaction force and the acceleration induced by the sum of all muscle forces to the acceleration on the whole body COM. Each line represents the resultant vector of the vertical and anterior-posterior acceleration caused by the ground reaction force (in grey) and the sum of all muscle forces (in black) a respectively. X and Y axis are scaled equally.

Models were evaluated according to the recommendations of Hicks et al., [34] to ensure possible sources of error were within acceptable tolerances. Scaled model dimensions, marker tracking errors, the influence of RRA on joint kinematics, trunk COM and residual forces and moments were evaluated across all simulations. Scale factors for pelvic width, depth and height were 1.07±0.06, 1.12±0.05 and 1.09±0.04 respectively. Mean peak RMS errors for scaling and tracking were 0.018±0.003 m and 0.021±0.015 m respectively. Mean residual pelvic forces and moments were all below 5% BW and 0.05 Nm/kg respectively (S1 Fig). Peak RMS error between residual reduced kinematics and experimental kinematics for RRA were below 2.5° across all DOF in all simulations (S2 Fig). The mean peak error between ground reaction forces and total contribution muscle forces was less than 5% body weight across all simulations (Fig 2). Passive muscle forces were found to be negligible (i.e. muscles tended to operate on the ascending limb and plateau region of the force-length relation).

### Statistical analysis

A between group Analysis of Variance was used to determine the effect of age group (young versus old) on step length and duration, trunk, pelvis and hip, knee and ankle joint angles for the stance and stepping limbs at each event (CR, TO, FC, KJM), 3D COM accelerations and individual contributions of each muscle to the 3D COM accelerations during balance recovery averaged over the periods defined from cable release to toe-off (CR-TO), toe-off to foot contact (TO-FC) and foot contact to knee joint maximum (FC-KJM). Statistical analysis was performed using the Statistical Package for the Social Sciences (Version 22, SPSS, USA) and significance was accepted for p<0.05.

## Results

### Effect of age on step length, step duration and joint angles

There were no significant age-related differences in step length (older adults: 0.83 ± 0.01 m; younger adults: 0.85 ± 0.01m), total step duration (CR-KJM) (older adults: 0.71 ± 0.1 s; younger adults: 0.72 ± 0.06 s) or the duration of any phase of the step recovery (older adults: CR-TO 0.21 ± 0.08 s, TO-FC 0.26 ± 0.01sec, FC-KJM 0.24 ± 0.04 s; younger adults: CR-TO 0.20 ± 0.04 s, TO-FC 0.27 ± 0.03 s, FC-KJM 0.25 ± 0.02 s). Further, no age-related differences in trunk, pelvis or stance or stepping limb joint angles were detected at CR, TO, FC or KJM.

### Effect of age on COM accelerations

**Fig 3:**
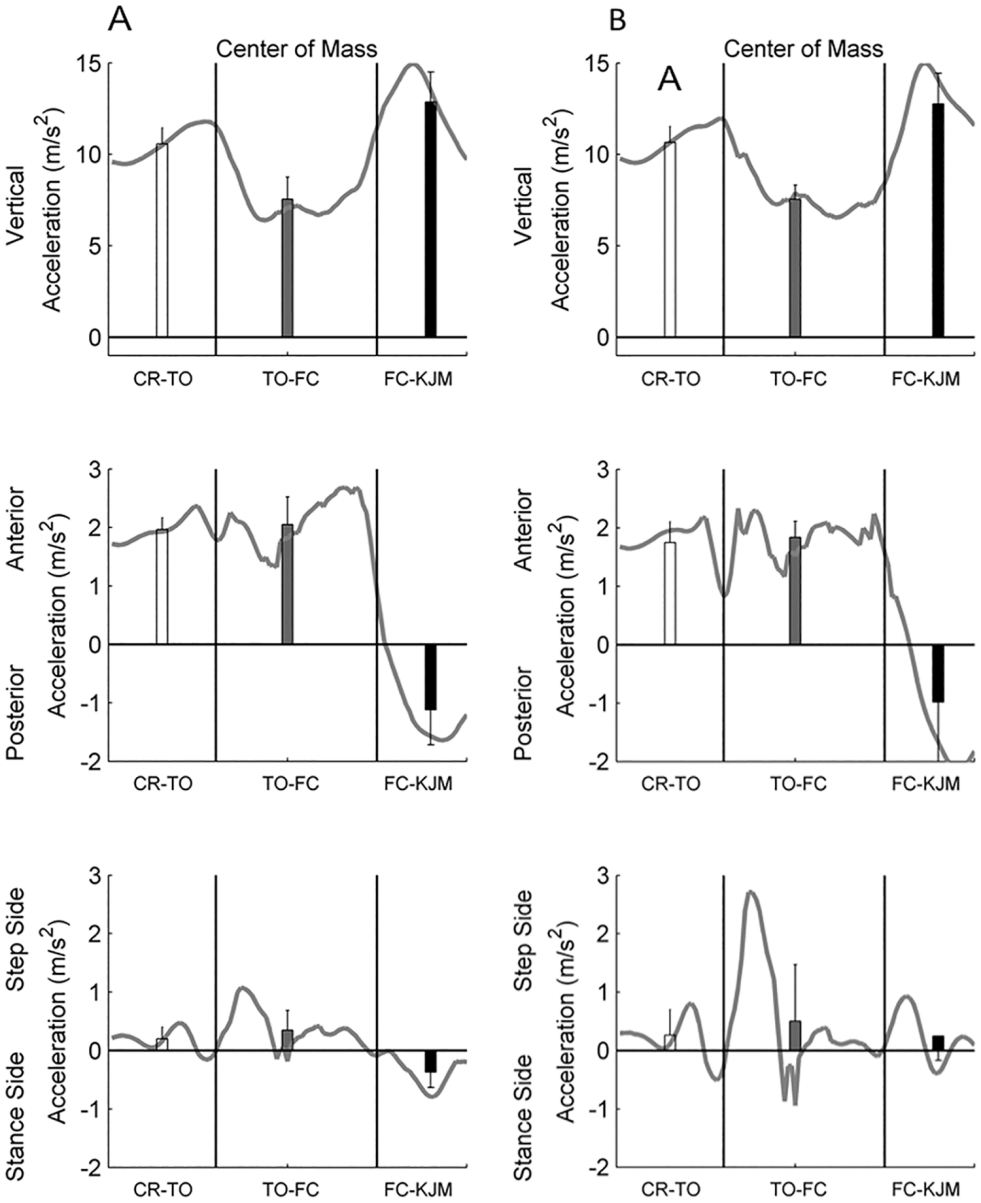
Instantaneous and mean COM accelerations for older adults (A) and younger adults (B) in the vertical, anterior-posterior and medial-lateral directions during balance recovery for the period from cable release and toe-off (CR-TO), toe-off to foot contact (TO-FC) foot contact to knee joint maximum (FC-KJM). Error bars represent one standard deviation.

There were no significant age-related differences in the 3D accelerations of the COM throughout balance recovery. For both groups, the COM experienced a net vertical acceleration throughout balance recovery, which was highest following FC. From CR to FC the mean horizontal COM accelerations for both groups were directed anteriorly and towards the step leg. Following FC, the anterior-poster COM acceleration was directed posteriorly (Fig 3).

### Effect of age on muscle contributions to COM accelerations

**Fig 4:**
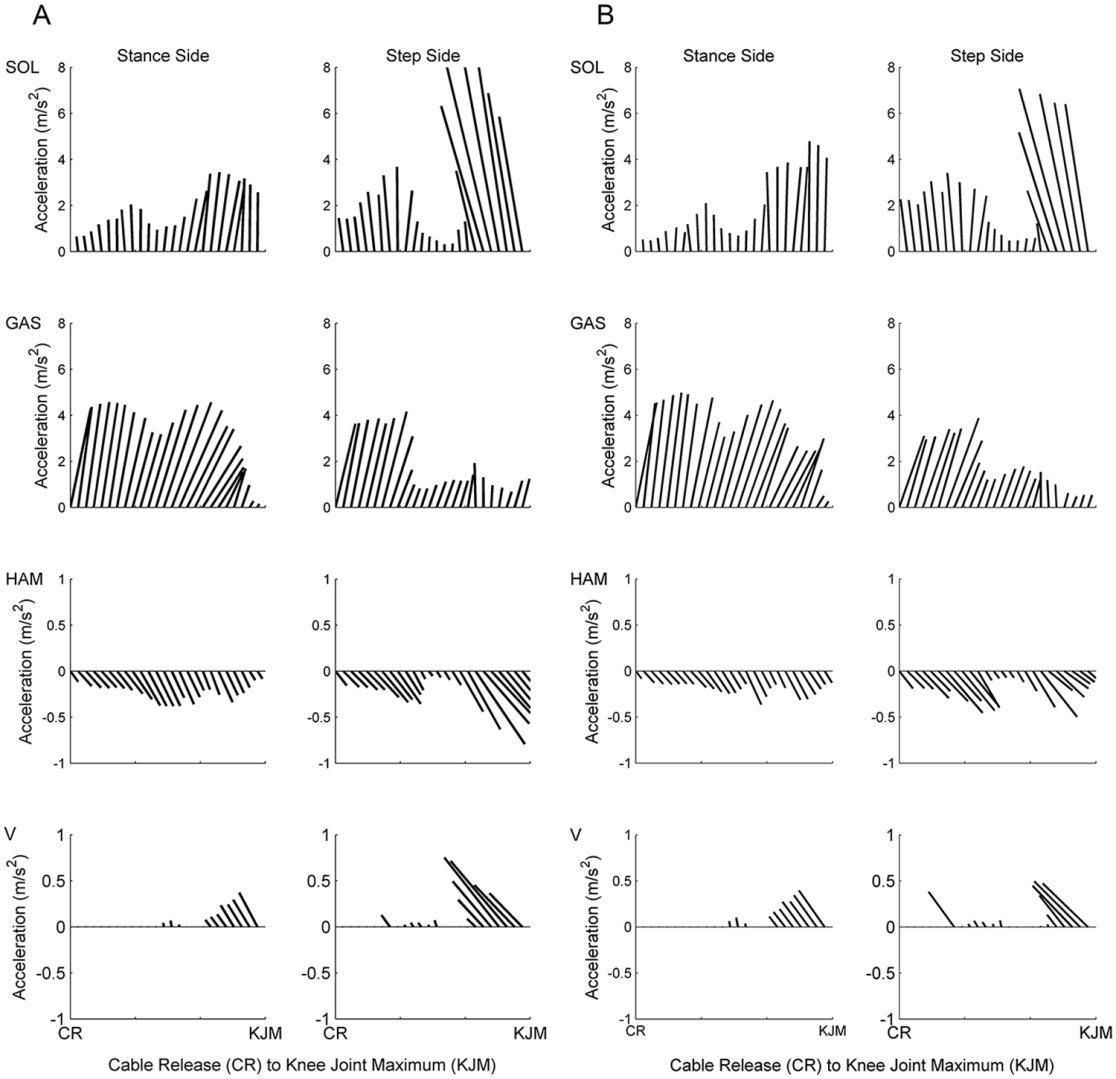
Vector plots representing the muscle induced accelerations of key muscles from the stance and stepping side limbs for older adults (A) and younger adults (B) from cable release (CR) to knee joint maximum (KJM) during balance recovery by stepping. Each line represents the resultant vector of the vertical and anterior-posterior acceleration induced by the respective muscle on the COM. The vertical and horizontal axis of each plot are scaled equally.

**Fig 5:**
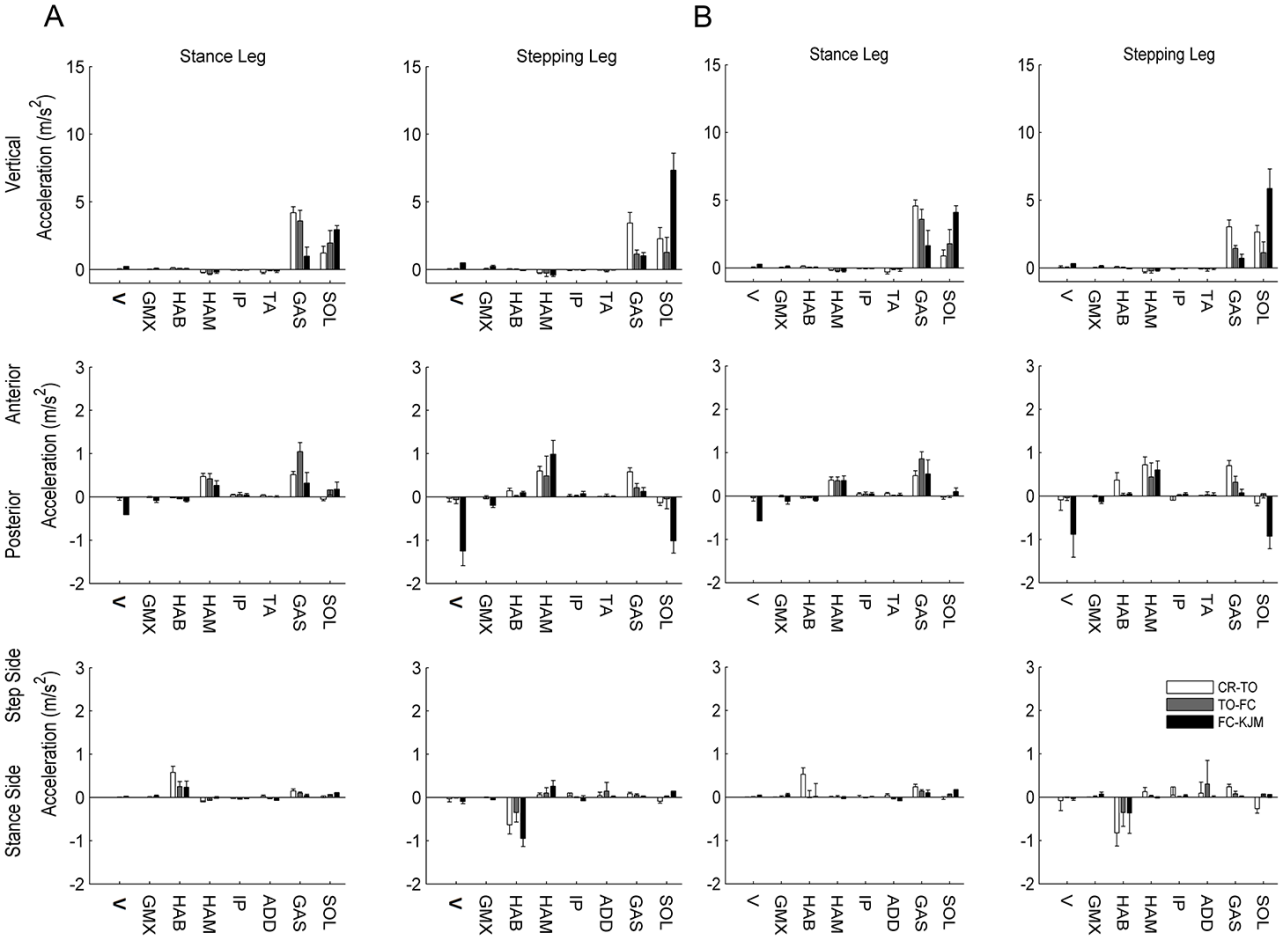
Mean muscle contributions to COM accelerations during balance recovery by stepping for older adults (A) and younger adults (B) from both stance side and stepping side muscles. Muscles presented are: Vasti (V), Gluteus Maximus (GMX), Hip Abductors (HAB), Hip Adductors (HAD), Hamstrings (HAM), Iliopsoas (IP) and Gastrocnemius (GAS) and Soleus (SOL). Error bars represent one standard deviation.

There were no significant age-related in the muscle contributions to the 3D accelerations of the COM. The main muscles responsible for accelerating the COM in the sagittal plane throughout balance recovery were the stance and step side Soleus, Gastrocnemius, Hamstrings and Vasti. The contributions of these muscles to the sagittal plane COM accelerations are depicted in Fig 4. Further, the contributions of all muscles to the 3D accelerations of the COM during each phase of balance recovery are displayed in Fig 5. The main muscles responsible for providing support were the stance and swing leg Gastrocnemius and Soleus. The main muscles responsible for the anterior acceleration of the COM prior to FC were the Gastrocnemius and Hamstrings of both limbs, whereas the main muscles responsible for posterior COM acceleration following FC were the stepping leg Soleus and Vasti. The stance leg Hip Abductors accelerated the COM towards the stepping side while the stepping side Hip Abductors simultaneously accelerated the COM towards the stance side throughout each phase of balance recovery.

## Discussion

This study found no age-related differences in the muscular control of recovery from forward loss of balance with a single step. These findings are consistent with studies that report a similar lack of difference in recovery mechanics between healthy young and healthy older adults [1, 27]. More pronounced differences in balance recovery mechanics have instead been reported between young adults and frail older adults [1, 4, 27, 35] and between young adults and older adults that require multiple compared to a single step to recover from the same lean magnitude [3, 15, 36, 37]. It was further demonstrated that the muscular control of successful recovery from forward loss of balance by stepping in both older and younger adults is achieved through interplay amongst key lower limb muscles which accelerate the COM forwards and towards the stepping limb during the stepping action, and cause a rapid deceleration of forward COM motion and acceleration of the COM towards the stance limb following touchdown of the stepping limb. The Gastrocnemius and Hamstrings on both limbs were the main muscles responsible for forward progression in the period from cable release to touchdown of the stepping leg. Following touchdown, deceleration of the COM was produced almost exclusively by the Soleus and Vasti muscles of the stepping leg. The net medial-lateral accelerations of the COM throughout balance recovery were relatively small compared to the other directions and were produced by the opposing actions of the stance and stepping leg Hip Abductor muscles. Gastrocnemius and Soleus muscles on the stance and stepping limbs were the largest contributors to the maintenance of support throughout balance recovery. Other muscles including the bilateral Erector Spinae, Gluteus Maximus, Iliopsoas, the Hip Adductors and Tibialis Anterior played a minimal role in accelerating the COM throughout recovery.

### Muscular control of anterior-posterior accelerations during balance recovery

The stance and stepping leg Hamstrings and Gastrocnemius muscles simultaneously accelerated the COM forwards throughout each phase of balance recovery and thereby opposed the braking action of the stepping leg Vasti and Soleus following foot contact. Although the forward-directed COM accelerations generated by the bilateral Hamstrings and Gastrocnemius might be interpreted as counterproductive to balance recovery, they are necessary for controlling the stepping action [27], and highlight the complexity of muscle coordination during balance recovery. The large contribution of the Vasti muscles to deceleration of the COM during balance recovery is consistent with findings for the early stance phase of walking [24] and running [22], where Vasti were reported to be the main muscle responsible for the deceleration experienced by the COM. However some differences were noted in the secondary muscles responsible for decelerating the COM in balance recovery compared to the early stance phase of walking and running. For example the rectus femoris, tibialis anterior and gluteus maximus contributed to deceleration of the centre of mass during early stance in running, whereas Soleus contributed to deceleration of the COM following foot contact in balance recovery. These differences are likely related to the much larger amount of trunk flexion that occurs during balance recovery [12, 13, 38] compared to walking and running where the trunk remains relatively upright. A longer recovery step and a more upright trunk have been consistently associated with greater ability to recover from forward loss of balance [9, 39]. In future it will therefore be of benefit to investigate muscle contributions to COM accelerations during balance recovery in older adults with balance impairments. Given that there is evidence that older adults can rapidly improve their balance recovery ability with repeated exposure to the task [11, 39], it would also be of benefit to understand how individual muscles contribute to these adaptations.

### Muscular control of medio-lateral accelerations during balance recovery

The Hip Abductors accelerated the COM in the opposite direction from their anatomical location throughout balance recovery (i.e. the stepping leg Hip Abductors accelerated the COM towards the stance side and vice-versa). Our results are in agreement with those reported for gait [23] and stair ascent and descent [26], which similarly indicted that ML accelerations of the COM are generated primarily through the Hip Abductor muscles. Although antagonistic contributions from the stepping and stance side Hip Abductors throughout balance recovery may appear inefficient, the greater metabolic energy expenditure is likely to be small [40] and of limited significance in the context of a balance recovery task. Because large relative muscle forces have been reported in the Hip Abductors during balance recovery [27], it is possible that poor Hip Abductor function may contribute to the deficits in medial-lateral stability reported in older adults [6] and could therefore be important muscles to target in future exercise-based fall prevention training studies.

### Muscular control of vertical accelerations during balance recovery

The vertical COM acceleration exceeded gravitational acceleration during the two periods of double support (i.e. from cable release to toe-off and following foot contact), and was below gravitational acceleration for the majority of the period of single leg stance from toe-off to foot contact. Gastrocnemius muscles of the stance and stepping limbs were primarily responsible for the provision of support of the COM from cable release to toe-off, with lesser contributions from the stance and stepping limb soleus. However following foot contact, the stepping limb Soleus became the dominant contributor to support with some lesser assistance from the Soleus muscle on the non-stepping (stance) leg. These findings are consistent with studies that demonstrate Soleus is the principal muscular source of support in the late stance phase of walking [24] and stance phase of running [22]. The smaller contribution of the Gastrocnemius to support following foot contact is likely due to its role as a knee flexor, which reduces its capacity to accelerate the COM vertically [22].

### Limitations

The results of this study should be considered with the following limitations in mind. Firstly, muscle force estimates and the accelerations they induce are sensitive to errors in the musculoskeletal geometry of the model. Errors associated with Scaling, IK and RRA were however kept within recommended tolerances [34] (see S1 and S2 Figs). Secondly, similar to modelling studies of gait [41, 42], muscle forces were estimated in the present study using a static optimisation approach that minimised muscle activation squared [43]. It is currently unclear to what extent this cost function reflects physiological behaviour of the system and so the findings from the present study might therefore be considered an initial estimate of how muscles contribute to recovery from forward loss of balance. The influence of the rigid tendon assumption of the muscle model on muscle force predictions and the extent to which the model adequately predicts measured muscle activation patterns, including co-contraction, will require further investigation. The finding of a reasonable correspondence between measured and modelled muscle activation patterns, as well as measured and modelled hip contact loads, in a prior study of balance recovery by our group [44] gives us some confidence in our current findings. In future it would also be of interest to evaluate alternative methods of predicting muscle force [45].

## Conclusion

This study demonstrated that the muscular control of the whole body centre of mass during single step recovery from forward loss of balance is similar for healthy young and older adults. Complex and sometimes opposing interactions of lower limb muscle forces are were used by young and older adults to control of the COM trajectory. The bilateral Gastrocnemius and Soleus were primarily responsible for providing vertical support, whereas the bilateral Hamstrings and Gastrocnemius accelerated the COM forwards throughout balance recovery and the stepping leg Vasti and Soleus decelerated the COM following foot contact. The stance and stepping leg Hip Abductors exerted opposing actions on the medio-lateral acceleration of the COM throughout the task. Deficits in the neuromuscular control of these key muscles could adversely influence recovery and should therefore be considered in interventions to improve balance recovery performance.

## Supporting information

S1 Fig Comparison of pelvic residual forces and moments derived from inverse dynamics (grey line) and the residual reduction algorithm (black line), plus reserve actuator moments from the lower limb of the stepping leg for a representative participant during balance recovery from cable release (CR) to knee joint maximum (KJM). (TIFF)

S2 Fig Comparison of joint angles derived experimetally from inverse kinematics (IK) and the residual reduction algorithm (RRA) for the lower limb of the stepping leg of a representative participant during balance recovery from cable release (CR) to knee joint maximum (KJM). (TIFF)
