# Supplementary figures and images for: Muscle contributions to the acceleration of the whole body centre of mass during recovery from forward loss of balance by stepping in young and older adults
Source: PLoS One. 2017 Oct 25;12(10):e0185564. doi: 10.1371/journal.pone.0185564 (PMC5656315; doi:10.1371/journal.pone.0185564)

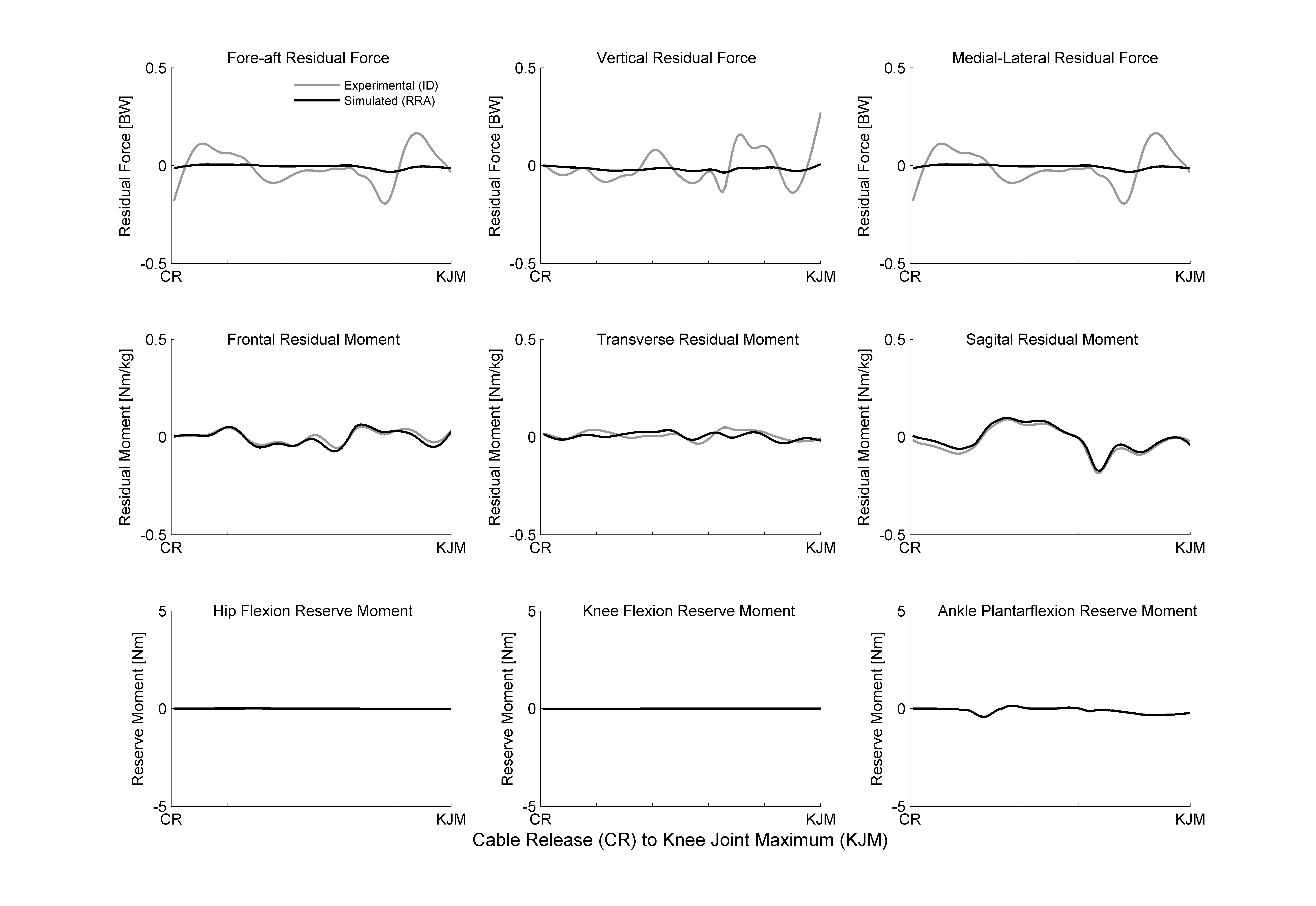

Supplement: S1 Fig — (TIFF) [file pone.0185564.s001.tiff]

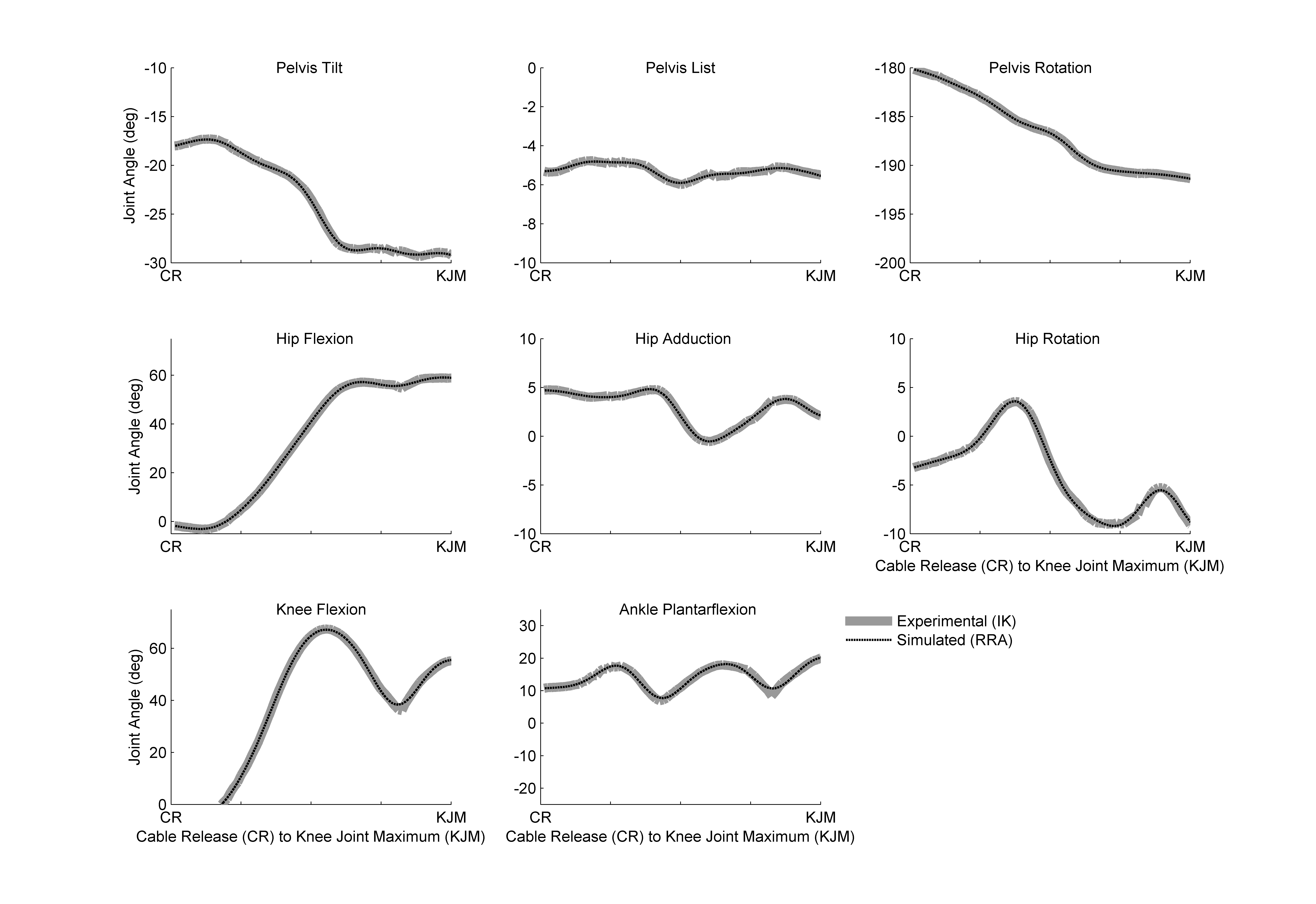

Supplement: S2 Fig — (TIFF) [file pone.0185564.s002.tiff]
